# *In Vivo* Function and Evolution of the Eutherian-Specific Pluripotency Marker UTF1

**DOI:** 10.1371/journal.pone.0068119

**Published:** 2013-07-09

**Authors:** Masazumi Nishimoto, Miyuki Katano, Toshiyuki Yamagishi, Tomoaki Hishida, Masayoshi Kamon, Ayumu Suzuki, Masataka Hirasaki, Yoko Nabeshima, Yo-ichi Nabeshima, Yukako Katsura, Yoko Satta, Janine E. Deakin, Jennifer A. Marshall Graves, Yoko Kuroki, Ryuichi Ono, Fumitoshi Ishino, Masatsugu Ema, Satoru Takahashi, Hidemasa Kato, Akihiko Okuda

**Affiliations:** 1 Radioisotope Experimental Laboratory, Research Center for Genomic Medicine, Saitama Medical University, Yamane Hidaka, Saitama, Japan; 2 Division of Developmental Biology, Research Center for Genomic Medicine, Saitama Medical University, Yamane Hidaka, Saitama, Japan; 3 Department of Anatomy and Cell Biology, Graduate School of Medicine, Osaka City University, Osaka, Japan; 4 Foundation for Biomedical Research and Innovation, 1-5-4 Minatojima-minamimachi, Chuo-ku, Kobe, Japan; 5 Department of Evolutionary Studies of Biosystems, The Graduate University for Advanced Studies (Sokendai), Hayama, Kanagawa, Japan; 6 Evolution, Ecology, and Genetics, Research School of Biology, The Australian National University, Canberra, Australian Capital Territory, Australia; 7 La Trobe Institute of Molecular Science, La Trobe University, Melbourne, Victoria, Australia; 8 Laboratory for Immunogenomics, RIKEN Research Center for Allergy and Immunology, Tsurumi-ku, Yokohama, Kanagawa, Japan; 9 Department of Epigenetics, Medical Research Institute, Tokyo Medical and Dental University, 2-3-10 Kandasurugadai, Chiyoda-ku, Tokyo, Japan; 10 Department of Anatomy and Embryology, Institute of Basic Medical Sciences, Graduate School of Comprehensive Human Sciences, University of Tsukuba, 1-1-1 Tennodai, Tsukuba, Japan; 11 Core Research for Evolutional Science and Technology (CREST), Japan Science and Technology Agency, Kawaguchi, Saitama, Japan; University of Connecticut, United States of America

## Abstract

Embryogenesis in placental mammals is sustained by exquisite interplay between the embryo proper and placenta. *UTF1* is a developmentally regulated gene expressed in both cell lineages. Here, we analyzed the consequence of loss of the *UTF1* gene during mouse development. We found that homozygous *UTF1* mutant newborn mice were significantly smaller than wild-type or heterozygous mutant mice, suggesting that placental insufficiency caused by the loss of *UTF1* expression in extra-embryonic ectodermal cells at least in part contributed to this phenotype. We also found that the effects of loss of *UTF1* expression in embryonic stem cells on their pluripotency were very subtle. Genome structure and sequence comparisons revealed that the *UTF1* gene exists only in placental mammals. Our analyses of a family of genes with homology to UTF1 revealed a possible mechanism by which placental mammals have evolved the *UTF1* genes.

## Introduction

During embryogenesis in placental (eutherian) mammals, cell division that gives rise to two cell lineages occurs around the formation of the blastocyst [1]. One of these constituents, the inner cell mass (ICM), gives rise to the entire embryo proper and some portions of the extra-embryonic tissues, while the other constituent, trophectoderm, is exclusively involved in the generation of extra-embryonic tissues. The placenta, one of the major extra-embryonic tissues, is a complex tissue comprising the allantoic/chorionic mesoderm, parietal/visceral endoderm, and chorionic ectoderm that is one of the derivatives of trophectoderm [2]–[4]. The placenta plays many important roles in nurturing the embryo, including the exchange of gases, nutrients and wastes between the embryo and its mother’s body. The placenta is also known to be an important source of hormones and growth factors involved in sustaining pregnancy.

Embryonic stem cells (ESCs) are established from the ICM, and are capable of growing indefinitely in culture while preserving their pluripotency [5], [6]. Since their discovery, ESCs have contributed greatly to progressing our understanding of embryonic development [7]. As a result of defining transcription factors that play critical roles in preservation of pluripotency, induced pluripotent stem cells (iPSCs) have been developed [8]. However, relatively little is known about the molecular mechanisms that underlie development of the placenta. The *Sox2* gene encodes one of the reprogramming factors used to generate iPSCs, which is strongly expressed in pluripotent early embryonic cells and extra-embryonic ectodermal cells (the immediate precursor of chorionic ectoderm), as well as in neural stem/progenitor cells. Moreover, gene targeting analyses have revealed a cell-autonomous requirement for Sox2 to preserve the undifferentiated characteristics of all three cell lineages that express *Sox2* [9]–[12]. The *UTF1* gene is widely recognized as one of the strict pluripotency marker genes, but its expression is not restricted to pluripotent embryonic cells. Similar to *Sox2*, *UTF1* is also expressed in extra-embryonic ectodermal cells but not in neural progenitor cells [13]. Recently, a new role of UTF1 in pluripotent cells has been uncovered, in which UTF1 limits bivalent gene silencing by preventing excessive PRC2 loading and H3K27 trimethylation [14]. However, the biological consequence of loss of *UTF1* in ESCs was not well defined in the previous study. Moreover, knockout mouse studies examining the roles of UTF1 in mouse development, including its role in other *UTF1*-expressing cells, i.e., extra-embryonic ectodermal cells, have not yet been performed.

Here, we used targeted disruption of the *UTF1* gene to examine its roles in mouse embryogenesis. We show that UTF1 is not essential for the entire process of embryogenesis. However, a *UTF1*-null background leads to a developmental delay in mid-gestation embryos and newborn mice. Our data suggest that placental insufficiency incurred by the loss of *UTF1* expression in extra-embryonic ectodermal cells at least in part contributes to this developmentally delayed phenotype. Genome sequence and structural analyses revealed that the *UTF1* gene exists only in placental mammals, and is not present even in the most closely related mammals (i.e. marsupials). Thus, we propose that *UTF1* has evolved to advance eutherian embryogenesis that strongly depends on placental function.

## Materials and Methods

### Construction of *UTF1*-targeting Vectors

Genomic clones carrying the *UTF1* gene were isolated from the mouse 129 SVJ λFixII library (Stratagene). The nucleotide sequence at the translation initiation site (5′-GGATGC-3′) was changed to provide a NcoI site (5′-CCATGG-3′) to facilitate subcloning. A targeting vector for the *UTF1* gene was designed to remove the region encoding from the 1^st^ initiating methionine to the 168^th^ amino acid to render the remaining region encoding from the 169^th^ to 339^th^ amino acid nonfunctional because of insertion of a drug resistance gene containing a stop codon, and a poly(A) addition signal preceded the remaining region. To construct targeting vectors, 5′ (2.9 kb BamHI-NcoI fragment) and 3′ (2.4 kb NotI-BglII fragment) homologous arms of the *UTF1* gene were cloned into a pBluescript (Stratagene)-based vector carrying the negative selection *TK* gene together with either the Neo-poly(A) cassette (Figure S1). The targeting vectors bearing either Bsd-poly(A) or puro-poly(A) cassettes were also constructed for electroporation-mediated sequential disruption of UTF1 loci (Figure S2).

### ESC Culture, Transfection, and Screening

To generate *UTF1* homozygous mutant ESCs by gene targeting, E14 ESCs were first adapted to a feeder-free condition as described by Niwa et al. [15]. Then, feeder-free cultured ESCs were transduced with the linearized targeting vector containing a blasticidin-resistance gene by electroporation, and then treated with 5 µg/ml blasticidin S. After selection of correctly targeted ESC clones, the linearized targeting vector containing a puromycin-resistance gene was transduced to disrupt the remaining wild-type allele of the *UTF1* gene. Southern blot and PCR analyses were performed using the probes and primers described in Figure S2A to identify *UTF1* homozygous mutant clones among ESC clones that were resistant to both blasticidin S and puromycin. To generate *UTF1* heterozygous mutant ESCs for blastocyst injection, the linearized targeting vector containing a neomycin resistance gene was transduced into E14 ESCs cultured on mitomycin C-treated MEFs. Correctly targeted ESC clones were then selected among G418-resistant clones by Southern blot analyses using the probes shown in Figure S1A.

### In vitro Differentiation of ESCs

To examine the differentiation propensity, *UTF1* heterozygous and homozygous mutant ESCs were subjected to embryoid body formation as described elsewhere [16]. RNAs were recovered from differentiation-induced or uninduced ESCs, reverse transcribed, and then analyzed by real-time PCR using Taqman probes according to the manufacturer’s instructions (Applied Biosystems).

### Immunostaining

Immunostaining was performed using the following primary antibodies at the indicated dilutions: anti-UTF1 (1∶100) [13], anti-phospho-histone H3 (Ser10) (1∶50) (Cell Signaling Technology), anti-Oct-4 (1∶400) (Santa Cruz Biotechnology), anti-Nanog (1∶200) (ReproCell), anti-nestin (1∶400) (Beckton-Dickinson), and anti-TujI (1∶500) (Convance). These antibodies were used in combination with appropriate Alexa Fluor dye-conjugated secondary antibodies (Molecular Probes). Cells were counterstained with DAPI (Sigma).

#### Establishment of iPSCs from MEFs and ESCs from blastocysts

iPSC induction was performed as described by Takahashi and Yamanaka [8] and establishment of ESCs from blastocysts was conducted according to a method by Bryja et al. [17], except under a feeder-free condition.

### Generation of *UTF1* Homozygous Mutant Mice

*UTF1* heterozygous mutant ESCs were injected into blastocysts to generate chimeric mice. Chimeras were bred to generate F1 heterozygous mutant mice that were backcrossed with wild-type female or male C57BL/6J mice over 10 generations before intercrossing between heterozygous mutant mice to generate *UTF1* homozygous mutant mice.

### Chimeric Mouse Analyses of *UTF1* Homozygous Mutant ESCs

*UTF1* homozygous mutant ESCs generated from blastocysts obtained by intercrossing between *UTF1* heterozygous mutant mice were labeled with fluorescent Kusabira orange protein. The labeled ESCs were injected into blastocysts that were transferred into surrogate mice. Embryos (9.5 dpc) were recovered and inspected under a fluorescence microscope as well as in bright field.

### Genomic DNA Data Analyses

All genomic DNA sequence data were collected from the Ensemble Genome Database and NCBI database. We noted that the opossum genome sequence data had two small gaps in the region subjected to our analyses, whereas the wallaby genome sequence lacked the entire portion. To fill in the two gaps present in the opossum assembly, oligonucleotide sets were designed to amplify these two gaps using available database information. These primers were used for PCR with the opossum genome as a template, and then the DNA sequences of the PCR products were determined. To obtain the wallaby sequence, BAC DNA (clone 411E1) bearing the *KNDC1* gene was pulled out from the Me_KBa (Arizona Genome Institute, USA) library panel using overgos designed for KNDC1 (5′-GTTTACTGTGTGGCAGCCATACTG-3′ and 5′-ATTTAGCTGCGGTCCACAG TATGG-3′). After confirmation of the presence of the *VENTX* gene in the BAC DNA by PCR and chromosomal location by fluorescence *in situ* hybridization on wallaby metaphase chromosomes as described by Deakin et al. [18], the entire DNA sequence was determined by shotgun sequencing. In this process, available opossum and wallaby genomic sequences were used as references for localization of the determined sequences. VISTA sequence alignment was performed using the VISTA program (http://www-gsd.lbl.gov/vista) based on the sequences obtained from the Ensemble Genome Database and NCBI database. For phylogenetic analyses, the sequences of *UTF1* (human, and mouse), *ZCAN20* (human, horse, elephant, cow, pig, opossum, and platypus), *ZSCAN20-like* (opossum, Tasmanian devil, and platypus) and *ZSCAN29* (human, and platypus) were aligned using Clustal W software with manual corrections. The number of nucleotide differences per site (*p*-distance) was then calculated using Molecular Evolutionary Genetics Analysis (MEGA) [19] for evolution analysis. A neighbor-joining tree analysis [20] was performed in MEGA using pairwise deletion for gaps/missing data.

### Accession Number

The accession number of the DNA sequence (BAC clone, 411E1 from the Me_KBa library) reported in this paper is AB731477.

### Animal Ethics

This study was carried out in strict accordance with the international and institutional guidelines. The protocol was approved by institutional review boards on the Ethics of Animal Experiments of the Saitama Medical University (Permit Number: 1020). All surgery was performed after the death by cervical dislocation under anesthesia with vapor of diethyl ether, and all efforts were made to minimize suffering.

### Supplemental Information

Supplemental Information includes seven figures and one table.

## Results

### Developmental Delay of the *UTF1* Knockout Embryo

**Figure 1:**
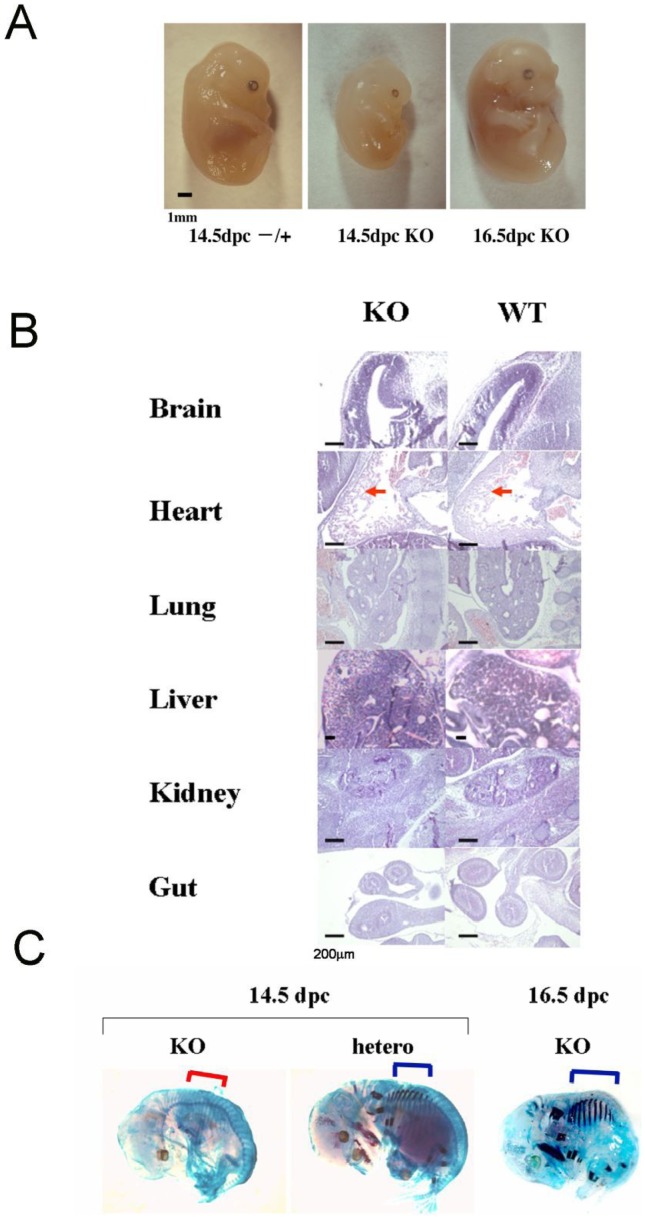
Growth retardation of *UTF1* homozygous mutant mouse embryos. (A) Macroscopic observation of *UTF1* homozygous embryos (14.5 and 16.5 dpc) and a heterozygous (14.5 dpc) embryo. (B) Histology of hematoxylin and eosin (H&E)-stained sections of various tissues from wild-type and *UTF1* homozygous mutant embryos at 14.5 dpc. Arrows indicate the myocardial layer. Scale bar corresponds to 200 µm. (C) Delayed maturation of the ribs in *UTF1* homozygous mutant embryos during embryogenesis. Alizarin and Alcian blue staining was applied to *UTF1* heterozygous and homozygous mutant embryos. Cartilaginous (14.5 dpc *UTF1*-null embryo) and ossified (14.5 dpc *UTF1* heterozygous mutant and 16.5 dpc *UTF1*-null mutant embryos) ribs are indicated by red and blue brackets, respectively.

To explore the role of the *UTF1* gene in development, we generated *UTF1* knockout mice (Figure S1). *UTF1* heterozygous mutant ESCs were used to produce chimeric mice. Heterozygous intercrosses yielded homozygous mutant embryos within the range of expected Mendelian ratios during gestational stages, suggesting that loss of UTF1 in mouse pluripotent cells does not eliminate their multi-lineage differentiation potential (Table S1). However, most of the *UTF1* homozygous mutant mice died within a day after birth and none survived for more than 2 days. Newborn *UTF1* homozygous mutant mice were significantly smaller than wild-type and heterozygous mutant mice at birth (Table S1). Growth retardation of *UTF1* homozygous mutant mice became evident at the mid-gestational stage (Figure 1A). However, a comparison between mutant embryos at different stages showed that they continued to increase in size, suggesting that this small phenotype does not reflect a blockade of development at specific embryonic stages, but is due to retardation of the developmental progression rate.

To detect any abnormality in the organs of *UTF1*-null mouse embryos, sections of wild-type and *UTF1* homozygous mutant embryos were prepared (Figure 1B). We observed no abnormalities in any tissue, except for the heart at 14.5 dpc, which was covered with a myocardial layer that was significantly thinner than that of wild-type embryos. However, by 16.5 days, the thickness of the myocardial layer of mutant embryos became equivalent to that of the wild-type embryo at 14.5 dpc (data not shown), which was consistent with the delay in the development process of mutant mice. We also used Alizarin and Alcian blue staining to demonstrate a delay in the replacement of cartilaginous ribs with ossified ribs in mutant embryos (Figure 1C). This observation can be also explained by the developmental delay in mutant embryos.

### Developmental Delay of the *UTF1* Homozygous Mutant Placenta

**Figure 2:**
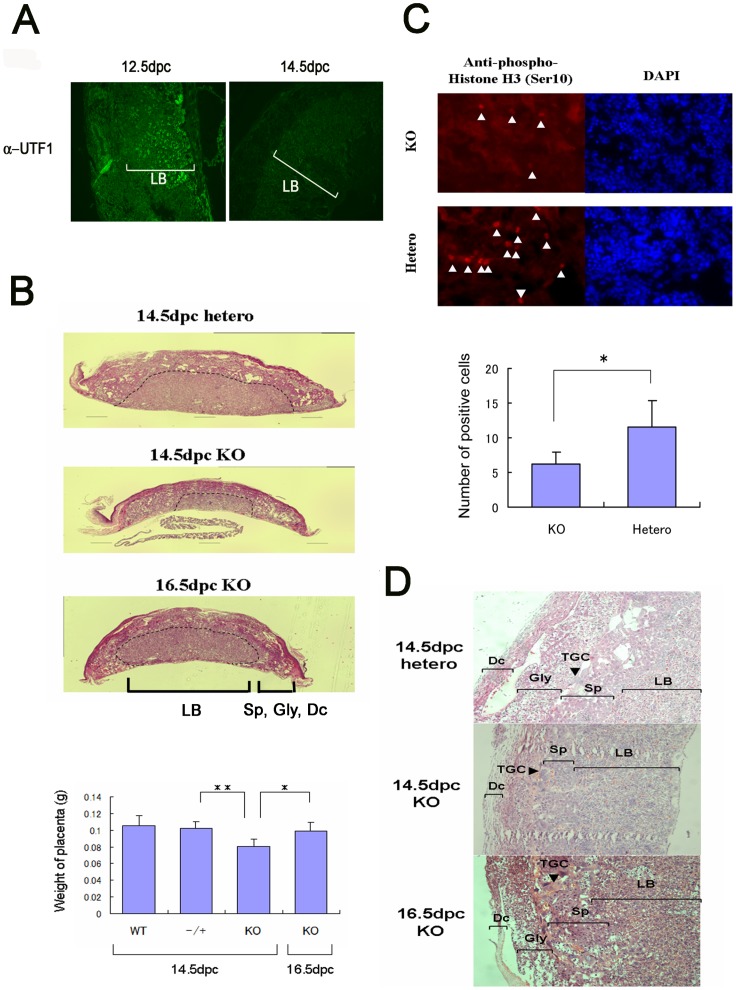
Role of UTF1 in the placenta. (A) Immunohistochemical analyses of UTF1 protein expression in wild-type placentas at 12.5 and 14.5 dpc. LB, labyrinth layer. (B) H&E-stained sections of *UTF1* heterozygous and homozygous mutant placentas. The boundary between the LB region and others is indicated by the dotted line. Each column of bar graph represents the mean of placenta weight with standard deviation (SD) (n = 3). *, p<0.05; **, p<0.01. (C) Cells in the mitotic phase in *UTF1* homozygous and heterozygous mutant placentas at 12.5 dpc visualized by immunostaining with an anti-phospho-histone H3 (Ser10) antibody. Phospho-histone H3-positive mitotic cells are marked by white arrowheads. Cells were counterstained with 4′,6′ diamidino-2-phenylindole (DAPI). Each column of bar graph represents the mean of number of phosphorylated histone H3 in 0.1 mm square with SD (n = 3). *, p<0.05. (D) Magnified view of H&E-stained sections of placentas. Placentas with the indicated genotypes were sectioned and stained. Dc, deciduas; Gly, glycogen trophoblast; TGC, trophoblast giant cell; Sp, spongiotrophoblast.

*UTF1* homozygous mutations provoke a developmental delay that becomes apparent at the mid-gestational stage. However, expression of the *UTF1* gene is abruptly silenced during gastrulation [13], and no *UTF1* expression is evident in mid-gestation embryos except for primordial germ cells (PGCs). We previously showed that *UTF1* is also expressed in extra-embryonic ectodermal cells as well as in pluripotent early embryonic cells [13]. Here, we also found that *UTF1* expression was readily detectable in wild-type 12.5 dpc placentas by immunohistochemical analyses, but UTF1 was undetectable at 14.5 dpc (Figure 2A). Furthermore, our data showed that UTF1 was expressed in trophoblast stem cells (Figure S3). Because hypotrophy of the placenta is one of the major causes of intra-uterine growth restriction syndrome [21], [22], a general delay in growth and development of homozygous null embryos can be explained by placental insufficiency. Consistent with this possibility, we found that the *UTF1* homozygous mutant placenta was significantly smaller than the wild-type placenta at 14.5 dpc, but continuously enlarged and, therefore, size of *UTF1* homozygous mutant placenta at 16.5 dpc was comparable to wild-type at 14.5 dpc (Figure 2B). The *UTF1* homozygous null placenta was also less mitotically active as revealed by analyses of phosphorylated histone H3 that marks mitotic cells (Figure 2C). Detailed histological inspection revealed the glycogen trophoblast cell layer in 16.5 dpc (but not 14.5 dpc) *UTF1* mutant placentas, demonstrating their equivalent developmental stage to that of 14.5 dpc wild-type embryos [23] (Figure 2D and Figure S4). Taken together, our results indicate that homozygous disruption of the *UTF1* gene results in a significant developmental delay of the placenta and embryo.

### Phenotypes of Homozygous *UTF1* Knockout Mice Depend on their Genetic Background

**Figure 3:**
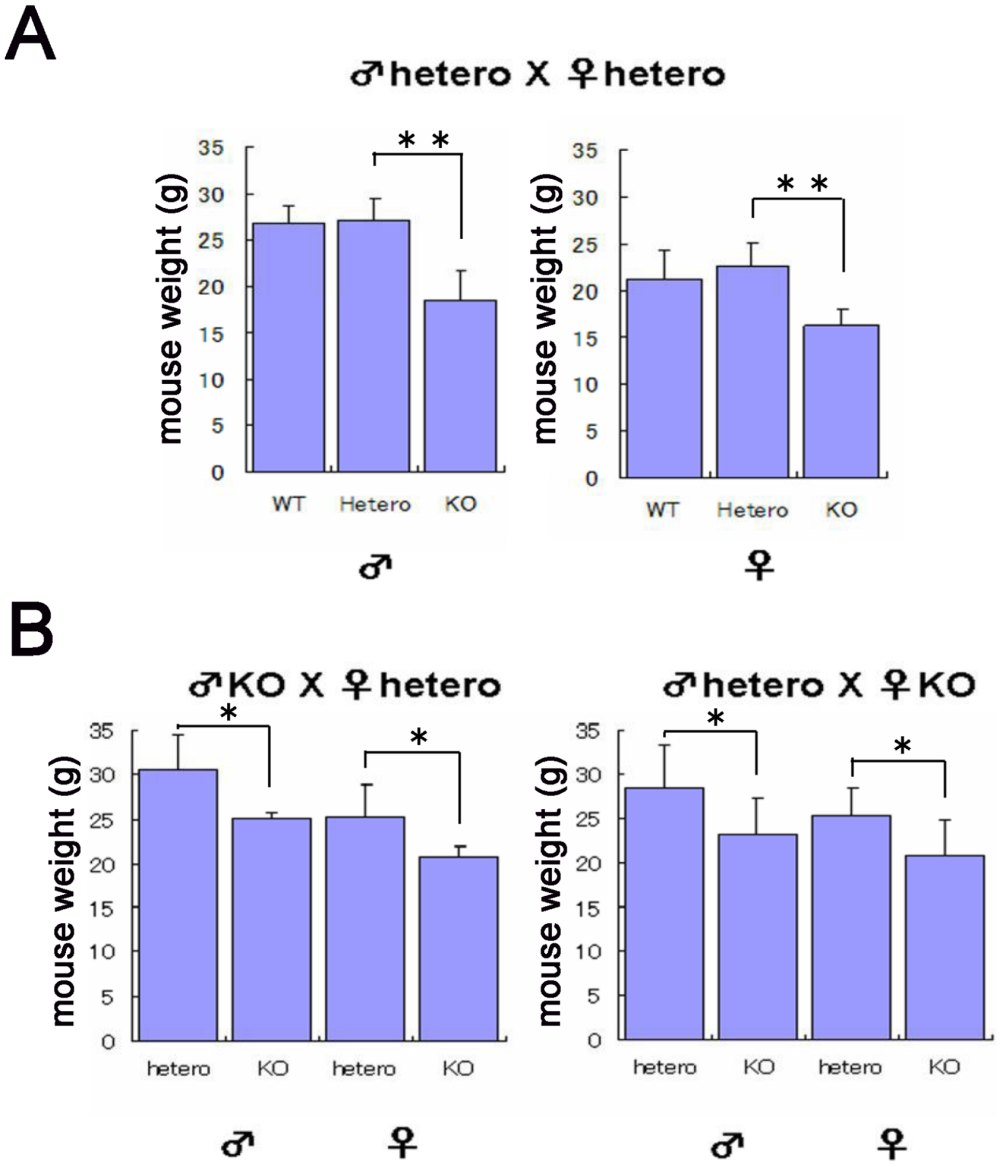
Phenotypes of *UTF1* homozygous mutant mice with a mixed genetic background of C57BL/6J (25%) and ICR (75%). (A) Weight of viable *UTF1* homozygous mutant mice generated by intercrossing heterozygous mutant mice that had been backcrossed twice with wild-type ICR mice. These analyses revealed that adult *UTF1* homozygous mutant mice (4-weeks-old) were viable, but significantly smaller than corresponding wild-type and heterozygous mutant mice. Data represent the mean with SD (n = 4). **, p<0.01. (B) Intercrosses between *UTF1* homozygous and heterozygous mutant mice. These intercrosses showed that the *UTF1* homozygous mutation with a mixed mouse genetic background did not affect fertility, but led to the generation of small mice. Mouse weights were examined at 4 weeks after birth. Data represent the mean with SD (n = 4). *, p<0.05;

It is known that the phenotypes associated with disruption of a gene sometimes vary significantly depending on the genetic background of the mice. The ICR mouse strain tends to show less extreme gene disruption effects than those of other mouse strains [24], [25]. We therefore intercrossed *UTF1* heterozygous mutant mice, which have a C57BL/6J background, with wild-type ICR mice and backcrossed the F1 progeny with ICR mice. We then used these *UTF1* heterozygous mutant mice with a C57BL/6J (25%) and ICR (75%) background to generate *UTF1* homozygous mutant mice. Surprisingly, we found that the *UTF1*-null mice generated after these procedures were viable and fertile, unlike *UTF1*-null mice generated from mice with a complete C57BL/6J background. However, growth defects in *UTF1* knockout embryos were equally evident irrespective of the genetic background of the mouse (Figure 3). These conspicuously milder phenotypes of *UTF1*-null mutants from mice with the mixed background are consistent with our hypothesis that *UTF1* knockout causes only a general delay of growth and development without leading to a specific defect.

### Acquisition of Pluripotency with the *UTF1*-null Background

**Figure 4:**
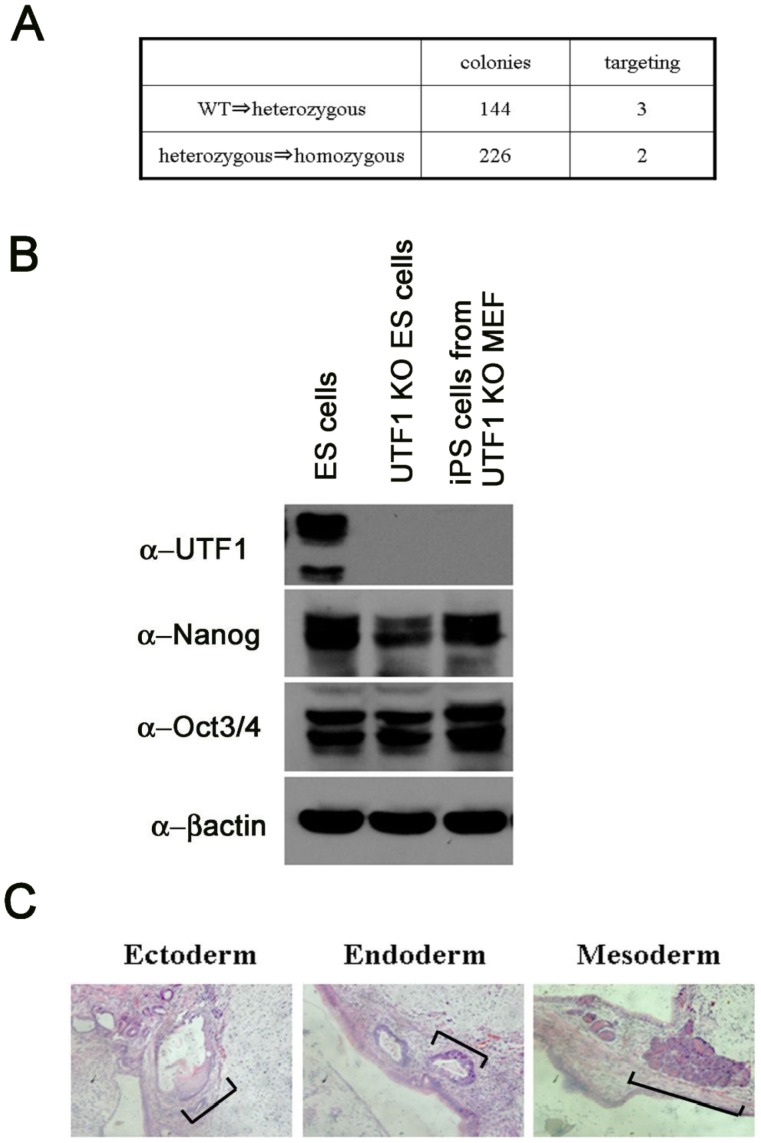
Acquisition of pluripotency by *UTF1*-knockout homozygotes. (A) Comparison of the frequency between knockouts of first and second alleles in serial *UTF1* gene targeting. A *UTF1-*targeting vector carrying the puromycin resistance gene was introduced into wild-type and *UTF1* heterozygous mutant ESCs bearing the blasticidin resistance gene in one of the *UTF1* loci by electroporation, and were then selected with medium containing puromycin only or puromycin and blasticidin, respectively. Genomic DNAs were prepared from drug-resistant clones and used as templates for PCR to distinguish between homologous recombination and random integration of the vector. Frequencies of targeted disruption in wild-type and *UTF1* heterozygous mutant ESCs were 2.08% and 0.88%, respectively. Because the probability of targeted disruption in wild-type ESCs is considered to be twice as high as that in heterozygous mutant ESCs, we concluded that the second allele knockout in serial gene targeting of the *UTF1* loci is not a rare event compared with the first allele knockout. (B) Western blot analyses of UTF1 and other pluripotency marker proteins in *UTF1* homozygous mutant ESCs (UTF1 KO ES) generated in A and iPSCs generated from *UTF1* homozygous mutant MEFs (UTF1 KO iPS). Wild-type ESCs were used as references. (C) H&E-staining of sections of a teratoma containing differentiated cells of all three germ layers generated by injection of *UTF1*-null iPSCs into nude mice. Representative portions of the three germ layers are marked by brackets.

Generation of viable mouse pups with the *UTF1*-null background was rather unexpected because UTF1 has been demonstrated to play important roles in pluripotency [14], [26] by analyses of ESCs. To scrutinize the roles of UTF1 in ESCs, we first examined whether there is any bias in generating *UTF1*-null ESCs from *UTF1* heterozygous mutant ESCs rather than generating heterozygous mutants from wild-type ESCs by electroporation-mediated disruption of the *UTF1* gene with the targeting vector (Figure S2). We found that at least no strong bias existed to disrupt the second *UTF1* allele compared with that of the first allele (Figure 4A). Nanog is one of the major pluripotency factors [27], [28], and is dispensable for maintaining, but essential for acquiring pluripotency [29], [30]. Therefore, we decided to explore the possibility of crucial involvement of *UTF1* in establishing pluripotency. To this end, we examined whether *UTF1*-null mouse embryonic fibroblasts (MEFs) can serve as somatic cells to generate induced pluripotent stem cells (iPSCs) by transduction of defined transcription factors [8], [31], [32]. Unlike *Nanog*-null somatic cells, we found that *UTF1*-null MEFs could generate iPSCs by retrovirus-mediated transduction of reprogramming factors. Consistent with previous reports [14], [26], we found that the expression levels of Oct3/4 and Nanog in *UTF1*-null ESCs and iPSCs were comparable with those in wild-type ESCs (Figure 4B). Moreover, we confirmed the ability of *UTF1*-null iPSCs to generate teratomas with all three germ layers (Figure 4C).

**Figure 5:**
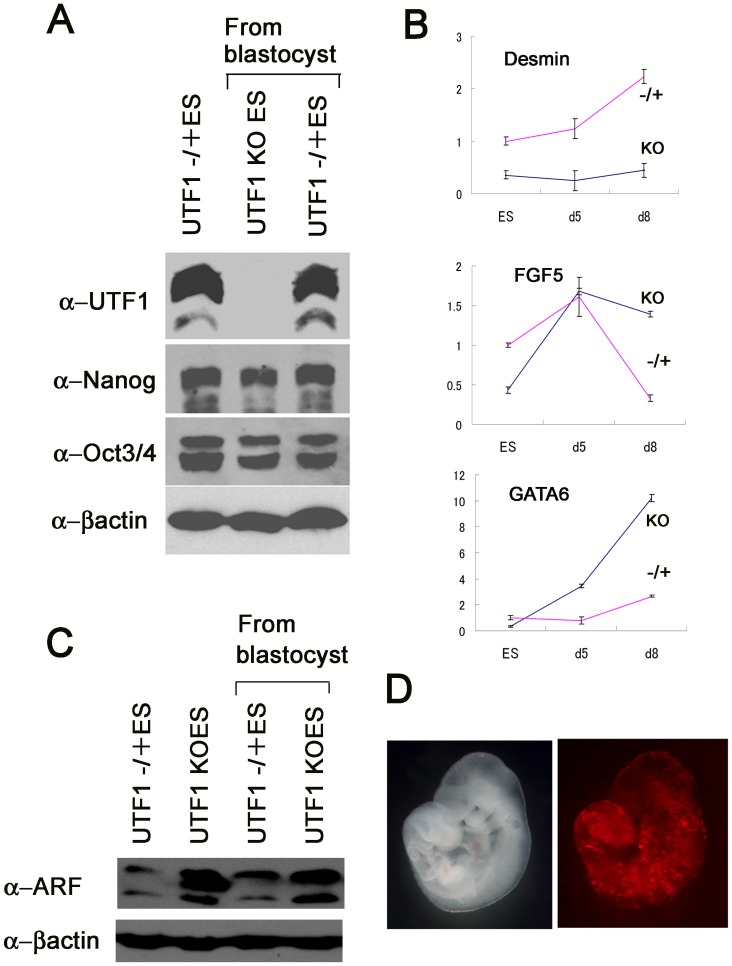
Isolation of UTF1 homozygous mutant ESCs from blastocysts. (A) Western blot analyses of pluripotency marker proteins in ESCs generated by outgrowth of *UTF1* homozygous and heterozygous mutant blastocysts. (B) Comparison of the differentiation propensity between *UTF1* homozygous and heterozygous mutant ESCs. *UTF1* homozygous and heterozygous mutant ESCs were induced to differentiate by embryoid body formation. RNAs were prepared at the indicated days. cDNAs generated by reverse transcription were used to examine the levels of differentiation marker gene expression by real-time PCR. Data represent the mean with SD (n = 3). (C) Western blot analyses of Arf tumor suppressor protein in *UTF1* heterozygous and homozygous mutant ESCs. (D) Chimeric mouse analyses of *UTF1* homozygous mutant ESCs. Fluorescent Kusabira orange-labeled *UTF1* homozygous mutant ESCs generated from a blastocyst were injected into blastocysts. Embryos were allowed to develop in a recipient female mouse. Left and right panels are bright-field and fluorescence images of a 9.5 dpc embryo recovered from the recipient mouse, respectively.

To determine whether *UTF1*-null ESCs can also be generated from *UTF1*-null blastocysts, we grew blastocysts obtained from *UTF1* heterozygous mutant intercrosses in ESC medium. Genotyping of 18 independent ESC clones revealed that five were null for *UTF1*, which was approximately the quarter of expected clones based on the Mendelian segregation ratio. The ESC status of these cells was again confirmed by the expression of Oct3/4 and Nanog (Figure 5A), and teratoma formation (data not shown). Thus, unlike the *Nanog* knockout, a *UTF1*-null knockout does not affect the establishment of ESCs or iPSCs. Examination of the differentiation propensity of *UTF1*-null ESCs confirmed the previous demonstration [14] of an alteration in the induction of differentiation due to the loss of UTF1, in which some differentiation marker genes became rather refractory to differentiation cues, while others underwent more extensive up-regulation compared with that in *UTF1* heterozygous mutant ESCs (Figure 5B). However, we did not observe noticeable difference between *UTF1* heterozygous and homozygous mutant of 7.5 dpc embryos with respect to expression levels of early differentiation marker genes (*brachyury* and *Cdx2*) (Figure S5). Also consistent with previous report [14], we found that Arf tumor suppressor protein levels was elevated in *UTF1* homozygous mutant ESCs compared to *UTF1* heterozygous mutants (Figure 5C). Next, as a rigorous test of pluripotency, we performed chimeric analyses using *UTF1*-null ESCs for blastocyst injection. Although we obtained chimeric 9.5 dpc embryos in which fluorescent Kusabira orange-labeled *UTF1*-null ESCs populated entire portions of an embryo, we could only obtain chimeric embryos in which contribution of *UTF1*-null ESCs was much less than that from ICM cells (Figure 5D). These results were rather unexpected because intercrosses of *UTF1* heterozygous mutant mice gave rise to viable, albeit small, *UTF1* homozygous mutant mice. One possible explanation is that *UTF1*-null ESCs bear an essentially appreciable, but not completely intact, pluripotent property, in which the defect becomes clearly discernible by competition with ICM cells for distribution in the embryo.

### UTF1 Gene was Generated in the Eutherian Radiation

The evolution of placental growth genes is of considerable interest because the longevity and complexity of the placenta in eutherians are the major characteristics that separate the sister groups of therian mammals. The *UTF1* gene has been identified in many eutherian mammalian species including mice [13] and humans [33]. To examine whether non-placental mammals also possess *UTF1*, we first searched for nucleotide sequences homologous to human *UTF1* in the available vertebrate genomes. No *UTF1* orthologues were found in fish, frog, lizard, bird, monotreme or marsupial genomes.

**Figure 6:**
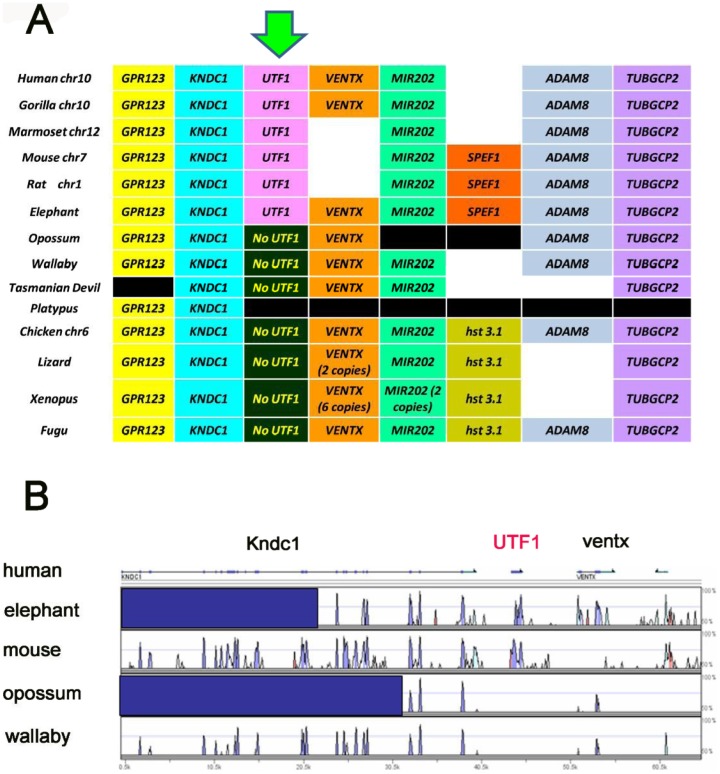
The *UTF1* gene is present only in the genomes of eutherian (placental) mammals. (A) Genomic organizations surrounding the *UTF1* locus in mammals and their corresponding genomic regions in other organisms. Black boxes indicate regions without available genomic sequences. (B) VISTA Browser (VGB2.0) plot of the 65.5 kb interval (ch10∶134,506,934-134,572,432) containing *KNDC1*, *UTF1* and *VENTX* genes in the human genome. Conservation plots for elephant (top panel) mouse (second panel), opossum (third panel) and wallaby (bottom panel), with respect to human, are shown in the coordinates of the human sequence (horizontal axis). Dark blue boxes indicate portions with unavailable DNA sequences in the database.

Next, we searched within these genomes for homologues of genes adjacent to *UTF1* in placental mammals. In eutherian genomes, *KNDC1, UTF1, VENTX* and *MIR202* genes were found in this order in which the *MIR202* gene resides closest to the end of the chromosome, although the *VENTX* gene is absent in marmoset and rodents. In marsupials, we found homologues to the *KNDC1–VENTX-mir202* syntenic region, but detected no sequences with significant similarity to *UTF1* (Figure 6A). The available opossum genome assembly has two small gaps in the region corresponding to the *UTF1* locus, and the wallaby genome has a large gap corresponding to the position of *UTF1* and adjacent genes. We resequenced to fill these gaps in both opossum and wallaby genomes, but found no sequences with similarity to *UTF1* (Figure 6B). We therefore conclude that *UTF1* was generated in the eutherian radiation.

**Figure 7:**
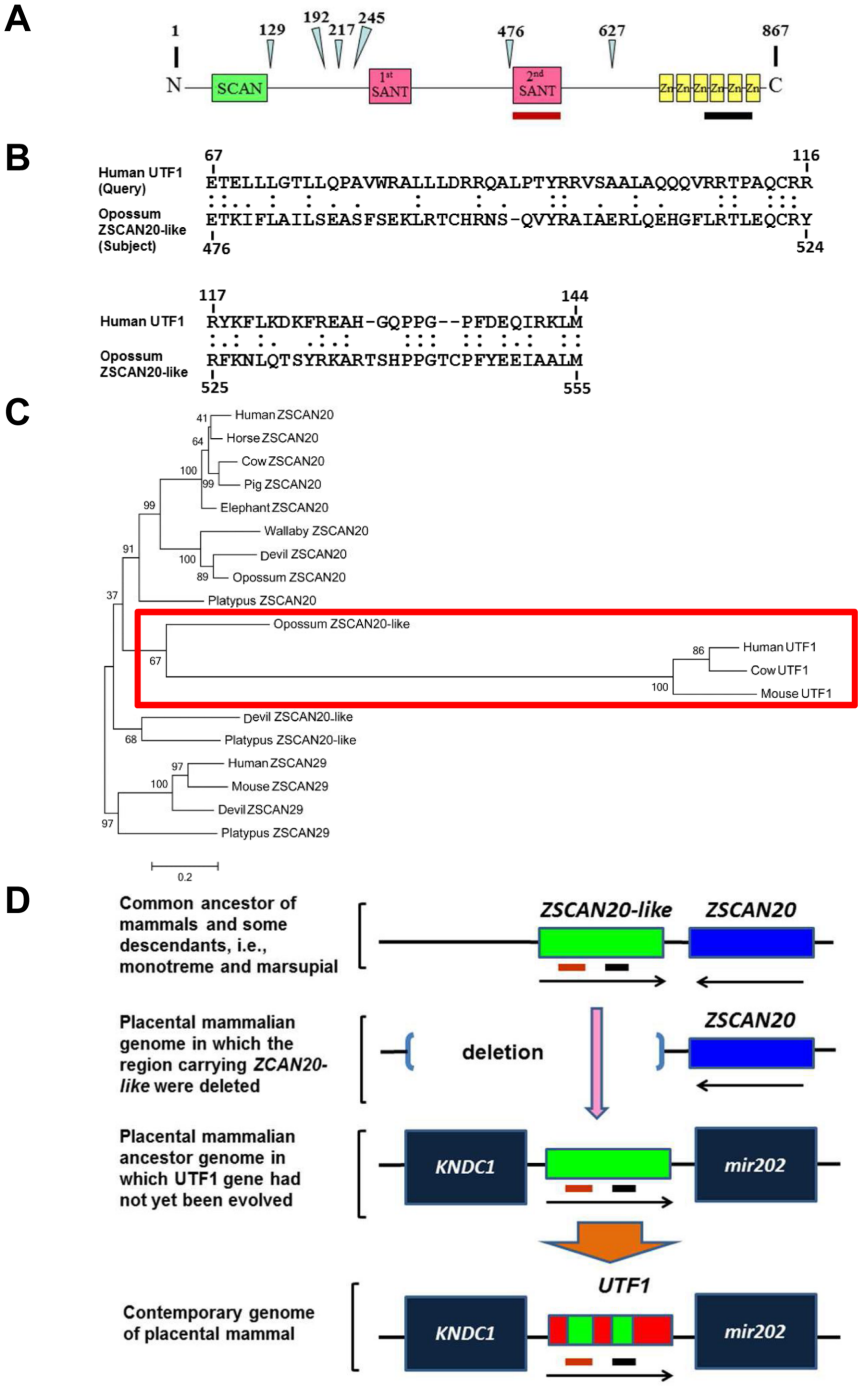
Implication of derivation of *UTF1* from *ZCSAN20-like* in an ancestor of placental mammals. (A) Diagram showing the domain structure of ZCSAN20-like protein with the amino acid sequence (1–867). Triangles with numbers indicate positions corresponding to exon-intron boundaries of the gene encoding ZCSAN20-like protein. Brown and black bars indicate positions with significant similarity to UTF1, which are shown in B and Figure S6, respectively. (B) Alignment of the amino acid sequence of the SANT domain of human UTF1 with that of ZSCAN20-like protein. Amino acid identity and similarity between UTF1 and ZSCAN20-like are marked by double and single dots, respectively. (C) A neighbor-joining tree analysis was constructed based on the number of nucleotide differences per site. The bootstrap value supporting each internal branch is indicated at the node. A cluster containing UTF1s and opossum ZSCAN20-like is boxed with a red line. (D) Model of *UTF1* evolution in a common ancestor of eutherian (placental) mammals after divergence which separates from marsupials. Data in B, C, and Figure S6 suggest that the *UTF1* gene was derived from a portion of the *ZCAN20-like* gene. After this transition, extensive nucleotide sequence changes occurred to evolve the *UTF1* gene, which eliminated the trace of homology between UTF1 and ZSCAN20-like except for two regions including their SANT domains. However, the depicted panel is just one possibility. For example, regions without similarity to the *ZCAN20-like* gene (depicted with a red rectangle) could have arisen *de novo* by modification of the sequences that had been present before translocation of the *ZCAN20-like* gene. The second SANT domain of ZSCAN20-like and its possible derivative in UTF1 are indicated by a brown bold line, while regions corresponding to those shown in Figure S6 are marked by a black line.

*UTF1* was possibly generated by *de novo* synthesis, or transposition from another genomic region. To examine the possibility of transposition from the ancestral sequence that gave rise to *UTF1*, we conducted an extensive search for *UTF1* homologs in the opossum genome. Blastx analyses revealed that the second SANT domain of *ZSCAN20-like* showed significant similarity (∼36% amino acid identity) to *UTF1* with an expected value of 4×10^–11^ (Figure 7B) (A diagram of domain structure of ZSCAN20-like protein is shown in Figure 7A). Furthermore, direct comparison of the *UTF1* sequence with that of the *ZSCAN20-like* gene revealed an additional region showing a similarity, which is located outside of the SANT domain, although it was not as strong as that found by the blastx analyses (Figure S6). Next, we examined the evolutional relationship between human UTF1 and ZSCAN20-like by phylogenetic tree analyses [20]. As a prerequisite step for those analyses, we generated a tree with respect to the amino acid sequences of all 96 different opossum proteins carrying the SANT domain, and found that ZSCAN2, 20, 20-like and 29 generated one cluster (data not shown). To further examine the relationship among them, a phylogenetic tree was generated with these four SANT domain-containing sequences, which showed that ZSCAN20 and ZSCAN20-like were tightly clustered as expected and ZSCAN29 was localized at the immediate outer-branch in the tree (Figure S7). Therefore, using ZSCAN29 as an out group sequence, we conducted phylogenetic tree analyses of UTF1 together with ZSCAN20, and ZSCAN20-like from several species. The results suggested a close relationship between UTF1 and opossum ZSCAN20-like with a high bootstrap value (89%) (Figure 7C).

## Discussion

UTF1 was originally identified as a transcriptional coactivator expressed in pluripotent early embryonic cells and extra-embryonic cells [13]. Because of its strong expression in pluripotent cells such as ESCs and the abrupt silencing of *UTF1* expression in pluripotent cells upon induction of differentiation, most investigations of *UTF1* have focused on its expression in pluripotent cells. For example, it has been shown that *UTF1* expression-based selection effectively eliminates contaminating differentiated cells from human ESC lines [34]. Pfannkuche et al. [35] used *UTF1* expression to improve the efficiency of obtaining genuine iPSCs with a pluripotency level equivalent to that of ESCs. Recently, new roles of UTF1 in pluripotent cells have been uncovered. UTF1 has been shown to prevent over-repression of bivalent genes by limiting surplus loading of the PRC2 complex, while UTF1 promotes degradation of mRNA transcribed from insufficiently repressed bivalent genes through recruitment of the mRNA decapping complex. UTF1 is also shown to be involved in ensuring a rapid growth rate by blocking Myc-Arf feedback control [14]. As described above, the roles of UTF1 in pluripotent cells are becoming more clearly depicted, but the *in vivo* function of UTF1 and the roles of UTF1 in extra-embryonic cells have not been investigated by knockout mouse studies so far.

Here, we report the establishment of homozygous *UTF1* mutant mice that survive at least up to birth. Thus, we showed that the *UTF1* gene is not absolutely essential for the multi-lineage differentiation potential of pluripotent cells *in vivo*. This observation may be the most significant result that we report here because numerous studies [14], [26], [36] have implied that UTF1 plays an essential function in early embryos. Although apparently milder phenotypes associated with *UTF1* gene knockout mice than expected could be explained by UTF1 homolog-mediated compensation, we consider that this may not be the case mainly because UTF1 sequence is very unique and genes showing significantly similarity to UTF1 are apparently not present in the genome. From a different point of view, we consider that our data showing that the *UTF1* gene is not an essential regulator, but a modifier of pluripotency for advancing eutherian embryogenesis are reasonable because chicken ESCs, which are devoid of the *UTF1* gene in their genome, have been shown to have properties that meet all the criteria of pluripotency, including the ability to re-enter development and contribute to the full repertories of somatic and germ cells in a recipient embryo [37], [38]. Although UTF1 is not essential for pluripotency, we found that *UTF1* homozygous mutation *in vivo* resulted in a significant developmental delay of the embryo, which became apparent during the mid-gestational stage. The *UTF1* gene was expressed in the placenta, but not in the embryo proper except for PGCs at the mid-gestational stage. Moreover, the *UTF1*-null placenta showed a growth defect. It is therefore conceivable that placental insufficiency caused by the loss of *UTF1* expression at least in part contributed to the developmentally delayed phenotype of *UTF1* homozygous mutant mice, albeit we cannot completely eliminate the possibility that *UTF1* gene disruption-mediated epigenetic changes and/or other changes occurred during early developmental stages contribute to apparent developmental delay of mid-gestation embryos. At present, we do not know the molecular mechanism of UTF1-mediated placental cell growth. It is possible that UTF1 supports cell proliferation in the placenta through blockade of Myc-Arf feedback control by destabilizing Arf mRNA, as has been demonstrated with ESCs [14].

Because of this developmental delay, newborn *UTF1* homozygous mutant mice with a C57BL/6J background did not survive for more than 2 days. However, we found that a mixed genetic background (C57BL/6J, 25%; ICR, 75%) completely eliminated this neonatal lethal phenotype, and the offspring, while small, were viable and even fertile. Although we do not know the basis for this effect of the genetic background, the normal yet small phenotype of *UTF1* homozygous mutant mice with the mixed genetic background further leads to the hypothesis that the *UTF1*-null genotype does not impose a specific detrimental abnormality, and only causes developmental delay.

Uterine nourishment is critical for embryogenesis in placental mammals [2], [3]. Although marsupials also have a fully functional placenta, a prominent feature of eutherian embryogenesis discriminating it from that in marsupials is the long gestational period [39], [40]. Therefore, it is possible that the *UTF1* gene is required to support placenta-dependent embryogenesis for a prolonged period in placental mammals. The small size of the *UTF1* gene (the mouse *UTF1* gene contains 1117 nucleotides from the initiating ATG to the stop codon, including an intron) and its conspicuously high sequence divergence (the overall identity of amino acids between mouse and human UTF1 is only 62%) are consistent with the recent emergence of *UTF1* in genome [41]–[45]. Consistent with this hypothesis, our genome structure and sequence comparison analyses revealed that the *UTF1* gene is indeed specific to placental mammals. There are other examples of recently evolved genes with a role in placental development, such as *PEG10* and *PEG11* [46]–[48]. Both of these genes are derived from an LTR-type retrotransposon; *PEG10* after the divergence of therian mammals which separates from monotremes and *PEG11* after the divergence separating from marsupials, although the ancestral retrotransposon from which it is derived is present in the marsupial genome.

Currently, the manner in which *UTF1* arose is unclear. Novel protein-coding genes arise either through sub-functionalization of pre-existing genes after gene duplication or by *de novo* synthesis from non-genic sequences [45], [49]. Our comparative analyses demonstrated that the second SANT domain of opossum ZSCAN20-like has significant homology with UTF1. Direct comparison between these two proteins revealed additional region of human UTF1 showing similarity to opossum ZSCAN20-like protein. Consecutive localization of *ZSCAN20* and *ZSCAN20-like* genes was found in the genomes of opossum and platypus (data not shown), which strongly suggests that these genes were most probably generated by tandem duplication. We assume that some physiologically important roles were conferred on *ZSCAN20* gene because of the high sequence conservation between numerous species including eutherian mammals. In contrast, the sequence of *ZSCAN20-like* gene is much less conserved between opossum and platypus compared to that of *ZSCAN20* gene. These facts indicate that the *ZSCAN20-like* gene sequence was subjected to extensive mutation because strong pressure of evolutional conservation was not subjected to this gene due to the presence of *ZSCAN20* gene. Furthermore, in conjunction with the similarity between human UTF1 and opossum ZSCAN20-like, the fact that eutherian genomes bear the *ZSCAN20* gene but lack the *ZSCAN20-like* gene suggests that the prototype or ancestral UTF1 sequence might be the *ZSCAN20-like* gene present in a common ancestor of placental mammals and marsupials. Ultimately, placental mammals may have evolved the *UTF1* gene at the expense of the *ZCAN20-like* gene. This evolutionary occurrence is similar to the mechanism by which the Prader-Willi imprinted gene *SNRPN* was generated from a copy of *SNRPB* that exists in marsupials, but not monotreme genomes [50].

Figure 7D shows our proposed model of the evolution of the placental mammal-specific *UTF1* gene. It is tempting to speculate that the evolution of the *UTF1* gene was one of the genomic innovations for advancing embryogenesis in the eutherian-specific manner.

## Supporting Information

Figure S1 **Generation of** ***UTF1*** **-null mice.** (A) Schematic representation of the *UTF1*-targeting strategy. Top: Restriction enzyme map of the mouse *UTF1* locus. Middle: Structure of the *UTF1*-targeting vector carrying *neomycin* (Neo)-resistance and *TK* genes for positive and negative selection, respectively. Bottom: Knock-in allele of the *UTF1*-targeting vector. I and II in black boxes indicate exons 1 and 2 of the *UTF1* gene, respectively. Red line corresponds to 7.6 kb BglII genomic fragment from wild-type *UTF1* locus, while magenta and blue lines correspond to 4.7 and 3.4 kb BglII/Nco1 genomic fragments from the targeted *UTF1* locus detected with 5′ and 3′ probes, respectively. The 3.4 kb BglII/Nco1 genomic fragment can be also detectable by the Neo probe. (B) Southern blot analyses of DNA from wild-type (WT) and *UTF1* heterozygous mutant ESCs (−/+). NcoI-BglII-digested genomic DNA was hybridized with the 5′, 3′ or neo probes shown in A. The expected size of the hybridized genomic DNA from wild-type and targeted *UTF1* alleles are indicated by arrows. Each bar color corresponds to genomic DNA fragment with the same color in A.
(TIF)

Figure S2 **Targeted disruption of the UTF1 gene in ESCs.** (A) Schematic representation of the UTF1-targeting strategy. A restriction enzyme map of the unmodified mouse UTF1 locus is shown at the top. The two subsequent rows show the structures of two UTF1-targeting vectors carrying either blasticidin S (Bsd) or puromycin (Puro) resistance genes. TK is the herpes simplex virus thymidine kinase gene used for negative selection. The bottom two rows show the UTF1 gene loci in which targeting vectors carrying either Bsd or Puro resistance genes were integrated by homologous recombination. The Genomic DNA fragments with red color or that of magenta in Figure S1A are also marked with the same colors. Green and orange lines correspond to 2.8 kb and 3.2 kb BglII-Nco1 fragments from knock-in loci of *UTF1* gene targeting vectors carrying Bsd and Puro resistant genes, respectively. (B) Southern blot analyses to distinguish among wild-type (WT), heterozygous (−/+) and homozygous (−/−) mutants with respect to the UTF1 loci. Each bar color corresponds to genomic DNA fragment with the same color in A. (C) PCR to detect the exact homologous recombination in ESCs with targeting vectors carrying either Bsd or Puro resistance genes. The primer sets used for PCR analyses are depicted in A and their sequences were as follows. UTF1 primer: 5′-ATGTGGCGCTCACTACTGCT-3′ Bsd primer: 5′-CCATGGCCAAGCCTTTGTCTC-3′ Puro primer: 5′-GAGCTGCAAGAACTCTTCCT-3′ Common primer: 5′-CAAACTGGTGAGGTTCGTTAAC-3′ (D) RNase mapping analyses of UTF1 RNA present in wild-type (WT), heterozygous (−/+) and homozygous (−/−) *UTF1* mutant ESCs. RNase mapping reactions were performed as described previously [16].
(TIF)

Figure S3 **Overlapping expression of UTF1 with the trophoblast stem cell marker Cdx2.** Trophoblast stem cells were established from ZHBTcH4 ESCs using fibroblast growth factor-4 according to a method by Niwa et al. [15]. Then, dual immunostaining of Cdx2 and UTF1 proteins was performed. These analyses revealed that most Cdx2-positive cells also expressed UTF1. UTF1 was also expressed in some Cdx2-negative cells, indicating that UTF1 shows relatively wider expression among trophoblast cell lineages than that of Cdx2.
(TIF)

Figure S4 **Lack of a glycogen trophoblast cell layer in the** ***UTF1*** **homozygous mutant placenta at 14.5 dpc.** H&E-stained sections of placentas shown in Figure 2D were further magnified and characteristic cell lineages (glycogen trophoblast, Gly; trophoblast giant cell, TGC; spongiotrophoblast, SP) in placentas with the indicated genotypes are shown. LB, labyrinth layer.
(TIF)

Figure S5 **Comparable expression of early differentiation marker genes in** ***UTF1*** **heterozygous and homozygous mutant embryos with extraembryonic tissues at 7.5 dpc.** Real-time PCR was conducted to quantitate expression levels of *brachyury* (T) and *Cdx2*. Expression level of each gene in *UTF1* heterozygous mutant was arbitrarily set to 1. Data represent the mean with SD (n = 3).
(TIF)

Figure S6 **Similarity in the amino acid sequence in addition to the SANT domain between UTF1 and ZSCAN20-like proteins.** Amino acid identity and similarity between UTF1 and ZSCAN20-like are marked by double and single dots, respectively. Amino acid identity is 29.5%.
(TIF)

Figure S7 **Neighbor-joining tree analysis of ZSCAN2, 20, 20-like and 29.** Sequences of ZSCAN2, 20, 20-like and 29 from several species were subjected to a neighbor-joining tree analysis. The analysis was performed as described in Figure 7C.
(TIF)

Table S1 **Analyses of pups obtained by** ***UTF1*** **heterozygous intercrosses.** Upper panel shows the results of genotyping analyses about pups obtained from *UTF1* heterozygous mutant intercrosses at the indicted days. Lower panel shows weights of new born mouse pups with the indicated genotypes.
(TIF)
