# Supplementary figures and images for: In Vivo Function and Evolution of the Eutherian-Specific Pluripotency Marker UTF1
Source: PLoS One. 2013 Jul 9;8(7):e68119. doi: 10.1371/journal.pone.0068119 (PMC3706607; doi:10.1371/journal.pone.0068119)

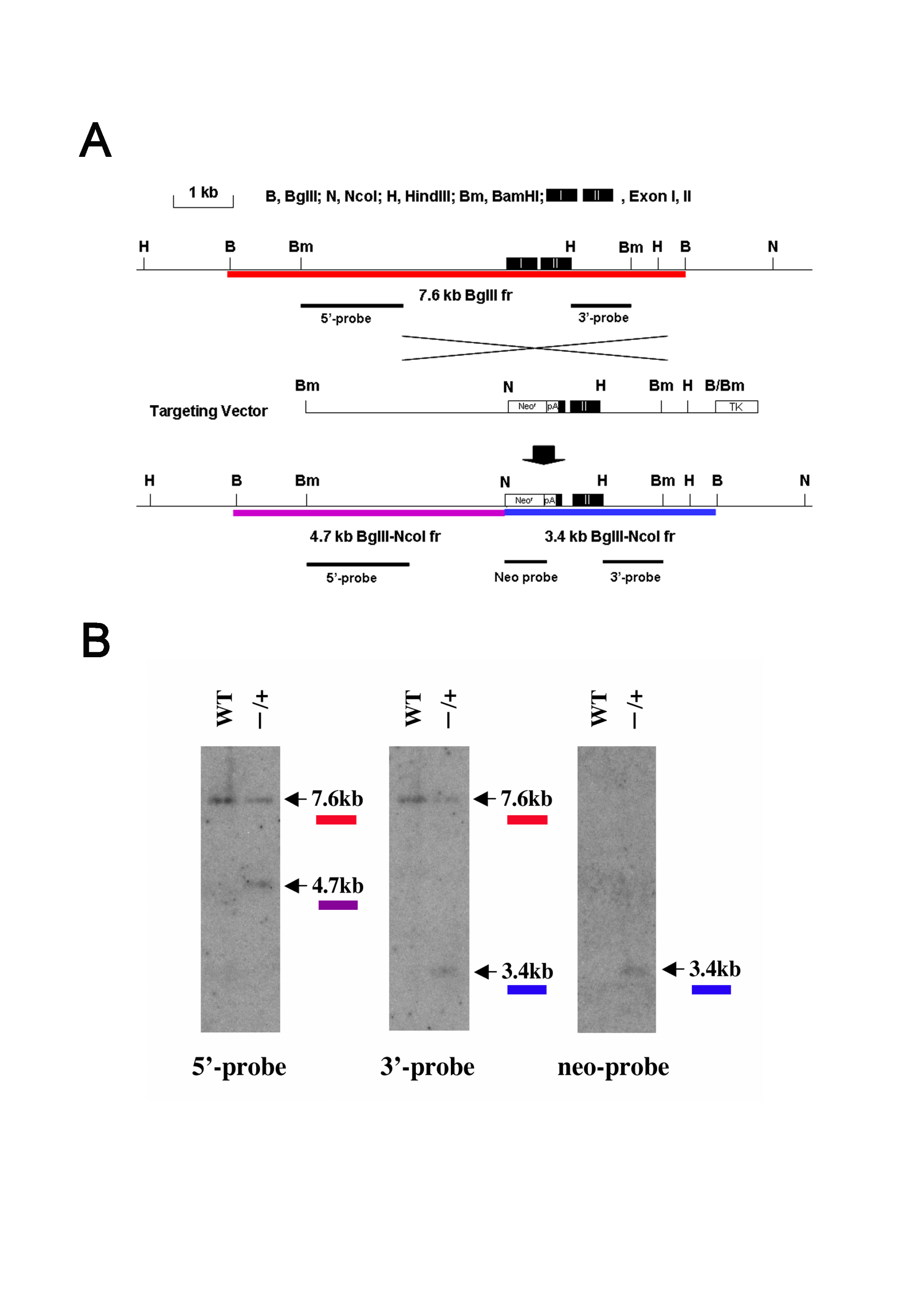

Supplement: Figure S1 — Generation of UTF1 -null mice. (A) Schematic representation of the UTF1-targeting strategy. Top: Restriction enzyme map of the mouse UTF1 locus. Middle: Structure of the UTF1-targeting vector carrying neomycin (Neo)-resistance and TK genes for positive and negative selection, respectively. Bottom: Knock-in allele of the UTF1-targeting vector. I and II in black boxes indicate exons 1 and 2 of the UTF1 gene, respectively. Red line corresponds to 7.6 kb BglII genomic fragment from wild-type UTF1 locus, while magenta and blue lines correspond to 4.7 and 3.4 kb BglII/Nco1 genomic fragments from the targeted UTF1 locus detected with 5′ and 3′ probes, respectively. The 3.4 kb BglII/Nco1 genomic fragment can be also detectable by the Neo probe. (B) Southern blot analyses of DNA from wild-type (WT) and UTF1 heterozygous mutant ESCs (−/+). NcoI-BglII-digested genomic DNA was hybridized with the 5′, 3′ or neo probes shown in A. The expected size of the hybridized genomic DNA from wild-type and targeted UTF1 alleles are indicated by arrows. Each bar color corresponds to genomic DNA fragment with the same color in A. (TIF) [file pone.0068119.s001.tif]

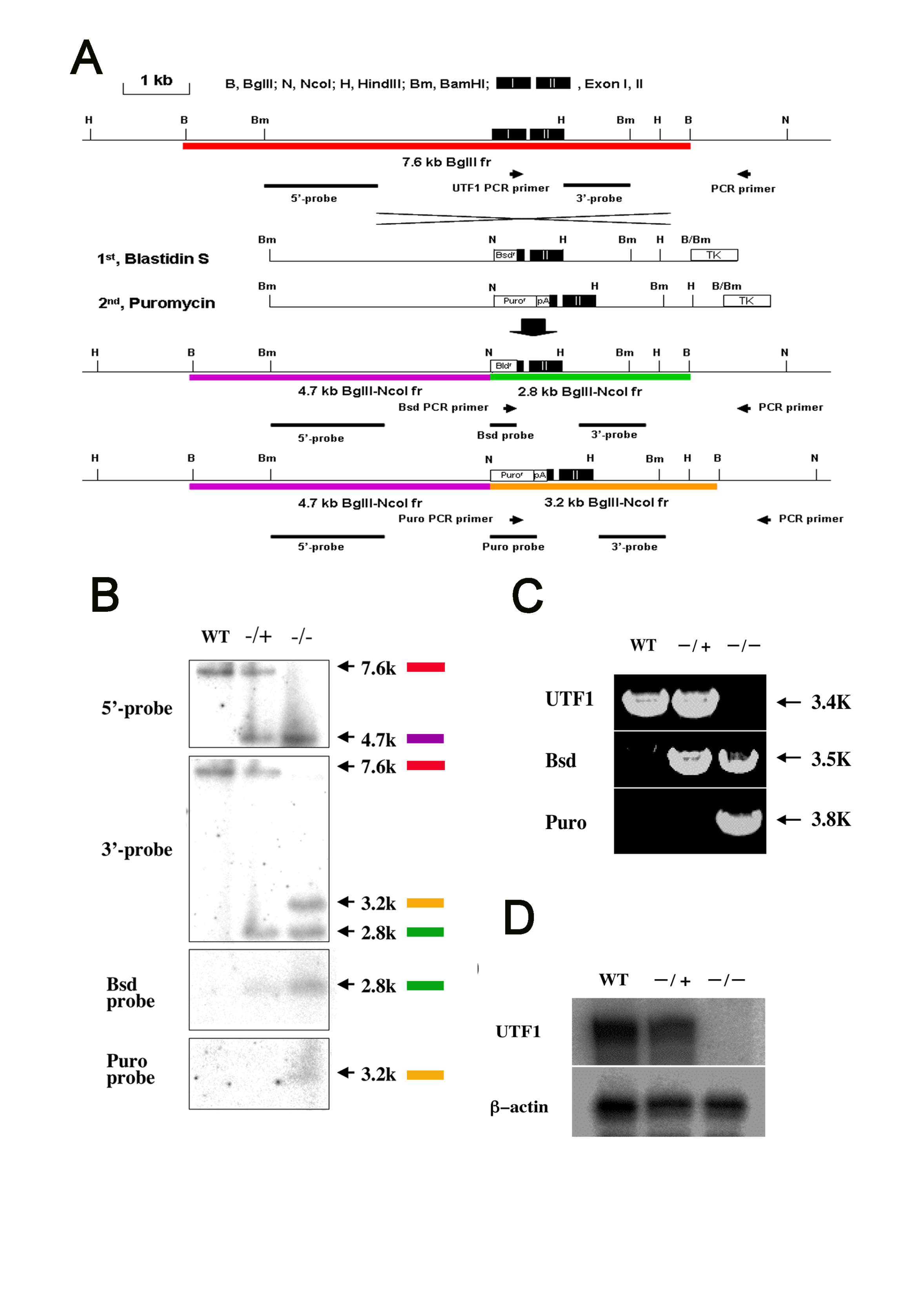

Supplement: Figure S2 — Targeted disruption of the UTF1 gene in ESCs. (A) Schematic representation of the UTF1-targeting strategy. A restriction enzyme map of the unmodified mouse UTF1 locus is shown at the top. The two subsequent rows show the structures of two UTF1-targeting vectors carrying either blasticidin S (Bsd) or puromycin (Puro) resistance genes. TK is the herpes simplex virus thymidine kinase gene used for negative selection. The bottom two rows show the UTF1 gene loci in which targeting vectors carrying either Bsd or Puro resistance genes were integrated by homologous recombination. The Genomic DNA fragments with red color or that of magenta in Figure S1A are also marked with the same colors. Green and orange lines correspond to 2.8 kb and 3.2 kb BglII-Nco1 fragments from knock-in loci of UTF1 gene targeting vectors carrying Bsd and Puro resistant genes, respectively. (B) Southern blot analyses to distinguish among wild-type (WT), heterozygous (−/+) and homozygous (−/−) mutants with respect to the UTF1 loci. Each bar color corresponds to genomic DNA fragment with the same color in A. (C) PCR to detect the exact homologous recombination in ESCs with targeting vectors carrying either Bsd or Puro resistance genes. The primer sets used for PCR analyses are depicted in A and their sequences were as follows. UTF1 primer: 5′-ATGTGGCGCTCACTACTGCT-3′ Bsd primer: 5′-CCATGGCCAAGCCTTTGTCTC-3′ Puro primer: 5′-GAGCTGCAAGAACTCTTCCT-3′ Common primer: 5′-CAAACTGGTGAGGTTCGTTAAC-3′ (D) RNase mapping analyses of UTF1 RNA present in wild-type (WT), heterozygous (−/+) and homozygous (−/−) UTF1 mutant ESCs. RNase mapping reactions were performed as described previously [16]. (TIF) [file pone.0068119.s002.tif]

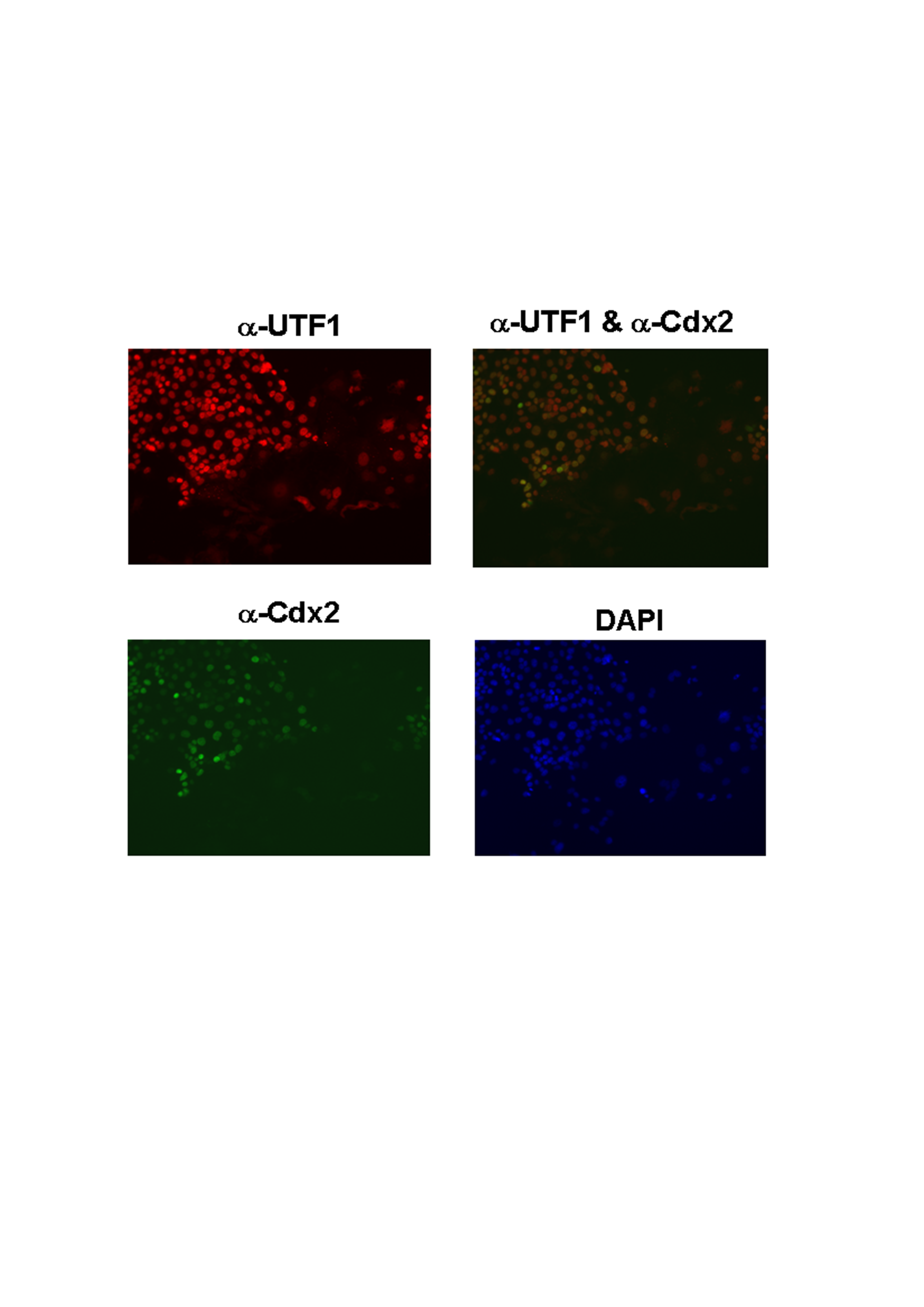

Supplement: Figure S3 — Overlapping expression of UTF1 with the trophoblast stem cell marker Cdx2. Trophoblast stem cells were established from ZHBTcH4 ESCs using fibroblast growth factor-4 according to a method by Niwa et al. [15]. Then, dual immunostaining of Cdx2 and UTF1 proteins was performed. These analyses revealed that most Cdx2-positive cells also expressed UTF1. UTF1 was also expressed in some Cdx2-negative cells, indicating that UTF1 shows relatively wider expression among trophoblast cell lineages than that of Cdx2. (TIF) [file pone.0068119.s003.tif]

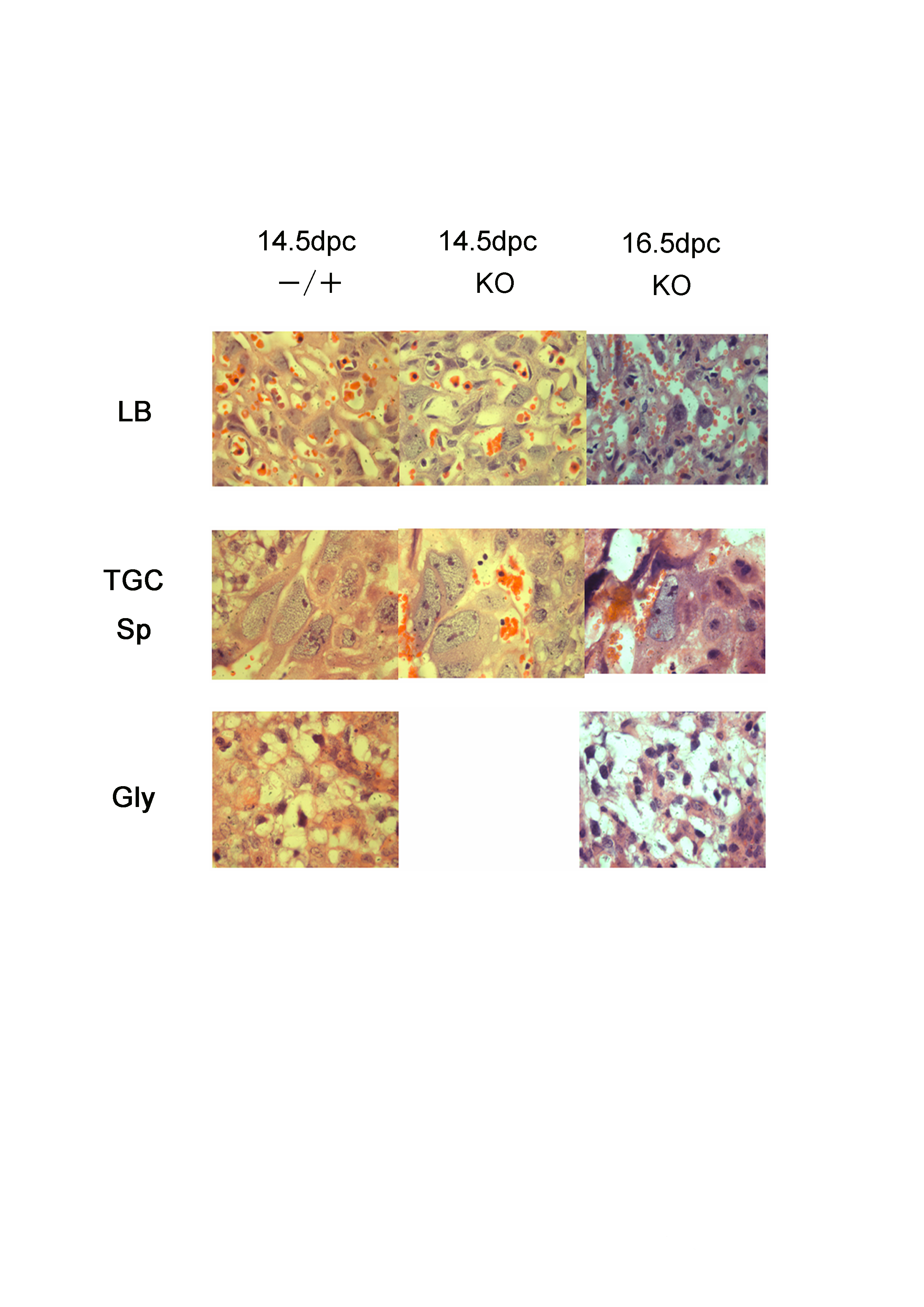

Supplement: Figure S4 — Lack of a glycogen trophoblast cell layer in the UTF1 homozygous mutant placenta at 14.5 dpc. H&E-stained sections of placentas shown in Figure 2D were further magnified and characteristic cell lineages (glycogen trophoblast, Gly; trophoblast giant cell, TGC; spongiotrophoblast, SP) in placentas with the indicated genotypes are shown. LB, labyrinth layer. (TIF) [file pone.0068119.s004.tif]

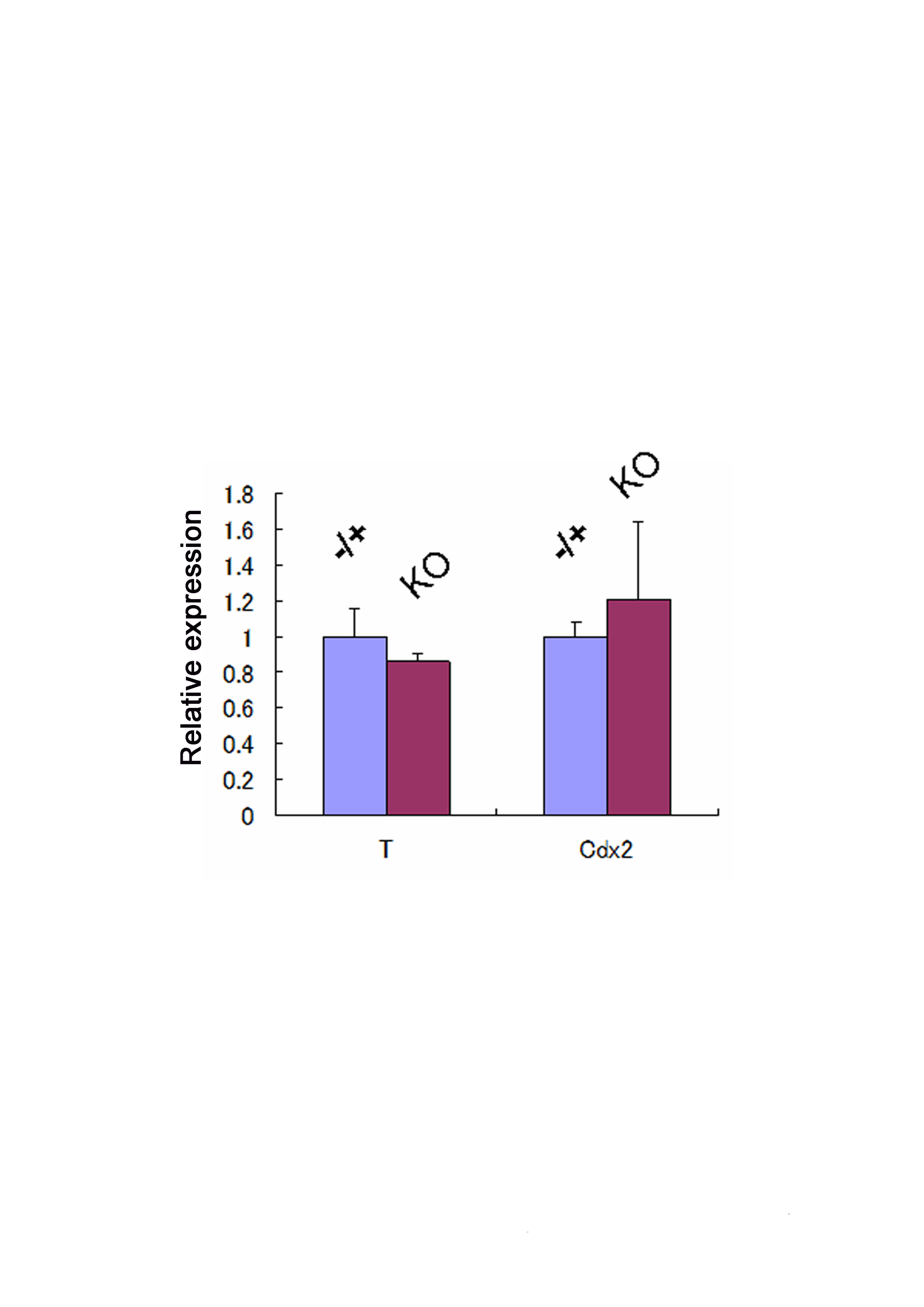

Supplement: Figure S5 — Comparable expression of early differentiation marker genes in UTF1 heterozygous and homozygous mutant embryos with extraembryonic tissues at 7.5 dpc. Real-time PCR was conducted to quantitate expression levels of brachyury (T) and Cdx2. Expression level of each gene in UTF1 heterozygous mutant was arbitrarily set to 1. Data represent the mean with SD (n = 3). (TIF) [file pone.0068119.s005.tif]

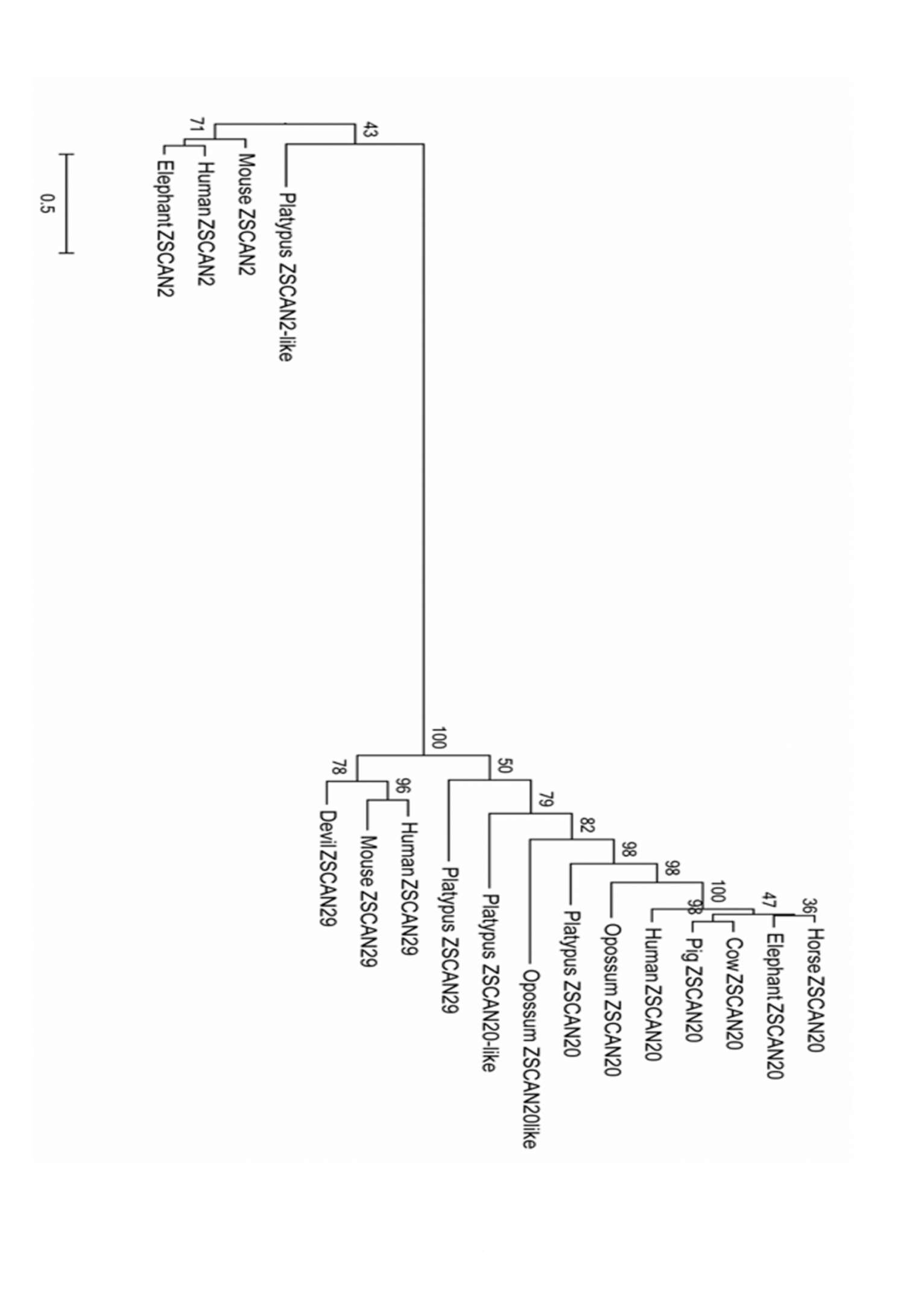

Supplement: Figure S7 — Neighbor-joining tree analysis of ZSCAN2, 20, 20-like and 29. Sequences of ZSCAN2, 20, 20-like and 29 from several species were subjected to a neighbor-joining tree analysis. The analysis was performed as described in Figure 7C. (TIF) [file pone.0068119.s007.tif]
